# Subconjunctival Bevacizumab Injection in Glaucoma Filtering Surgery: A Case Control Series

**DOI:** 10.1155/2013/384134

**Published:** 2013-03-14

**Authors:** Jing Wang, Paul Harasymowycz

**Affiliations:** ^1^Department of Ophthalmology, Maisonneuve-Rosemont Hospital, 5415 Boulevard de l' Assomption, Montreal, QC, Canada H1T 2M4; ^2^Ophthalmology Service, Department of Surgery, Centre Hospitalier Université de Sherbrooke, Hôtel Dieu, 580 Bowen Sud, Sherbrooke, QC, Canada J1G 2E8

## Abstract

*Aims*. To describe the use of subconjunctival bevacizumab (SCB) injection in the combined cataract and glaucoma filtering surgery (GFS). *Methods*. Retrospective comparative case series. Thirty eyes of twenty-eight patients who had GFS followed by SCB injection as part of post-operative management were included (Group SCB). The types of GFS included trabeculectomy and non-penetrating glaucoma surgery (NPGS) with mitomycin-C. Outcome measures included the reduction of intraocular pressure (IOP) and medications. Age-matched patients who had the same types of surgery without SCB were selected as a control group (Group C). *Results*. The types of GFS were: combined cataract surgery and NPGS (SCB: 20; C: 24), phacotrabeculectomy (SCB: 6; C: 3), NPGS (SCB: 3; C: 2) and trabeculectomy alone (SCB: 1; C: 1). The average follow-up time was 16.9 (±8.2) months in the SCB group and 19.6 (±11.5) months in the controls. 1.25 mg of bevacizumab was injected on average 14.1 (range: 3–42) days post-GFS. The mean IOP decreased from 21.9 (±9.8) to 11.9 (±4.7) mmHg in the controls and from 19.6 (±8.9) to 14.0 (±4.7) mmHg in the SCB group. There was no statistically significant difference between the two groups (*P* = 0.11). Complications included three cases of branch vein occlusion in the SCB group. *Conclusions*. SCB did not result in better outcome in term of IOP reduction. Clinicians should monitor its side effects in glaucoma patients.

## 1. Introduction

A key determinant of a successful glaucoma filtering surgery (GFS) is the healing response of the sclera, Tenon tissue, and conjunctiva. Histologically, functional blebs show low inflammatory response with scattered conjunctival epithelium cells and fibroblasts, whereas dysfunctional blebs are characterized by dense collagen tissues and corkscrew blood vessels [1]. Agents that modify the healing process have been used to improve the success of GFS. Antiproliferative agents such as 5-fluoroacil (5-FU) and mitomycin-C (MMC) inhibit the proliferation of fibroblasts. Both intraoperative use and postoperative use of these two agents have significantly improved the success of GFS, especially in high-risk patients [2]. However, the use of these antiproliferative agents can lead to very thin blebs that are prone to leakage, hypotony, and higher incidence of endophthalmitis [3, 4]. Other modulating agents such as growth factors inhibitors have been investigated for their potential use in GFS. Among these, antitransforming growth factor-*β*_2_ (TGF-*β*_2_) has shown promising results in experimental studies but has failed to show any superiority to placebo group in clinical trial [5].

In addition to inflammation and fibroblast proliferation, angiogenesis also plays an integral role in the wound healing process. The level of vascular endothelium growth factor (VEGF) has been shown to be upregulated in the aqueous humour of human and rabbit eyes after trabeculectomy [6]. However, the use of antiangiogenic agents in GFS has not been explored extensively. Case series have shown that postoperative subconjunctival injection of bevacizumab (SCB) decreased the bleb vascularity and IOP [7]. At the time of writing, three published prospective studies have evaluated the adjunct use of SCB in trabeculectomy and phacotrabeculectomy for patients with open angle glaucoma (OAG) [8–10]. The study by Grewal and associates is a prospective noncomparative case series of twelve trabeculectomies augmented with SCB (1.25 mg) immediately at the end of the surgery. None of their patients had previous ocular surgery. MMC and 5-FU were not used. At six months, successful outcome (IOP between 6–16 mmHg) was achieved in eleven out of the twelve eyes. In their study, blebs increased their vascularity after 3 months. The authors hypothesized that the use of SCB might decrease the incidence of cystic thin blebs, frequently noted after MMC augmented trabeculectomies [8]. The study by Nilforushan and associates is a randomized comparative study comparing the uses of SCB and MMC in trabeculectomy surgery [10]. Thirty-six eyes were randomized to receive either SCB (2.5 mg) or MMC (0.02% for 3 minutes). None of the eyes had previous ocular surgery. The followup was about seven months. The eyes that received SCB had a statistically higher IOP level compared to the eyes that received MMC (13.6 ± 3.2 mmHg versus 9.6 ± 2.7 mmHg). In their study, no difference in bleb appearance was noted between the two groups. The study by Sengupta and associates is a randomized study comparing the uses of MMC (0.03% for 3 minutes), direct intraoperative bevacizumab application on the scleral using a sponge and three doses of SCB (1.25 mg) in patients undergoing phacotrabeculectomy [9]. In their study, the patients randomized to the SCB group received two doses of SCB during the surgery and the third dose at one week. At six months, they reported a higher rate of success (IOP less than 18 mmHg) in the SCB group compared to those of the other two groups (90% versus 60%). All the three studies have relative short followups. Considering the current evidence, we intend to examine the use of SCB in the postoperative management of combined cataract and glaucoma surgery including both trabeculectomy and nonpenetrating glaucoma surgery (NPGS).

## 2. Patients and Methods

The study was a retrospective review of patients who had glaucoma filtering surgery (GFS) and subconjunctival bevacizumab injection (SCB) as part of their postoperative management. All patients have given written informed consent to receive SCB. All surgeries and postoperative followups were managed by one of the authors (P. Harasymowycz). The study subjects were identified through searching a computerized internal medical database by typing the keywords such as “subconjunctival bevacizumab,” “trabeculectomy,” and “nonpenetrating glaucoma surgery.” A control group was selected matching the demographic data and surgical procedures of the SCB group. Subjects from the control group received similar postoperative management as the SCB group with the exception of subconjunctival bevacizumab injection. Demographics data, types of GFS, intraocular pressure (IOP) and the number of topical IOP-lowering medications used pre- and postsurgery, the frequency and timing of SCB injections, other interventions used in the postoperative period, and complications were recorded. 

### 2.1. Glaucoma Filtering Surgery

The types of GFS included trabeculectomy and NPGS with or without phacoemulsification and posterior chamber intraocular lens implantation (PCIOL). In both GFS procedures, a conjunctival incision was made 1.5 mm posterior to the limbus, and a half thickness 3.5 × 3.5 mm scleral flap was dissected toward the limbus. In NPGS, a second deeper flap was dissected and excised, leaving an anterior trabeculodescemet window and the anterior wall of Schlemm's canal unroofed. In trabeculectomy surgery, a sclerostomy was created using a Kelly punch, and a peripheral iridectomy was performed with Vannas scissors. In both cases, MMC (0.2 mg/mL) was routinely applied for 2 minutes on the bare sclera followed by thorough irrigation of conjunctival tissues with balanced salt solution, the scleral flap was closed with interrupted 10.0 nylon sutures, and the conjunctiva was closed using 10.0 Biosorb using the modified Wise technique. In combined GFS and cataract extraction, the anterior chamber was entered under the scleral flap with a 2.75 mm keratome, and phacoemulsification was performed using a direct chop technique followed by cortical clean-up and implantation of a foldable intraocular lens. At the end of surgery, topical moxifloxacin was applied, and 2 mg of subconjunctival dexamethaosone was injected.

### 2.2. Postoperative Management of Bleb

All patients received topical moxifloxacin four times per day for one week, nonsteroidal anti-inflammatory four times per day for one week, and topical steroid tapering off for over two months. 

During the postoperative follow-up period, all patients could have the following interventions at the surgeon's discretion: subconjunctival injection of 5-fluorouracil (5-FU), laser suture lysis, bleb needling with a 30-gauge needle with or without 5-FU, laser goniopuncture, and subconjunctival injection of betamethasone or triamcinolone acetonide. Subconjunctival 5-FU injection and needling were performed when excess scarring was observed. 5-FU (5 mg/0.5 mL) was injected superiorly and adjacent to the bleb. Needling was performed using a 30-gauge needle tip to the flap area and then beneath the flap to puncture episcleral fibrous tissue. The needle tip was passed several times beneath the flap until bleb elevation was observed. Following the needling, subconjunctival injection of 5-FU (5 mg/0.1 mL) was occasionally used during the same session depending on the clinical appearance of the bleb. Scleral flap sutures were lysed using an argon green laser or red laser (0.1 s, 400–420 mW, 50 um spot size). Goniopunctures with Nd:Yag laser (4 mJ) were performed using a Latina gonioscopy contact lens. 

In addition to the previously mentioned interventions, all patients in the SCB group also received at least one dose of SCB postoperatively. SCB was given when the bleb showed marked vascularization with corkscrew vessels. After topical anesthesia, 1.25 mg (0.05 mL) of bevacizumab was injected in the subconjunctival space superior to the bleb using a 30-gauge needle. 

### 2.3. Outcome Measurements

Main outcome measurements included IOP and topical medication use recorded on the last clinical visit. Any complication during the follow-up period was also noted. Statistical analysis was performed using SPSS software version 16 (SPSS, Inc., Chicago, IL, USA).

## 3. Results

The database retrieved 30 eyes of 28 patients who had SCB injection following either trabeculectomy or NPGS in postoperative period. Matching for age, diagnosis, and surgical procedures, we selected 28 control patients who did not received SCB as part of their postoperative management. Demographic data and postoperative management for each group are shown in Tables 1 and 2. Most of patients were Caucasians and had open angle glaucoma. Age, preoperative IOP, and medications used were not significantly different in each group. The mean follow-up time was 19.6 (±11.5) and 16.8 (±8.2) months for the control and SCB groups, respectively. Four types of surgery were observed in the series. The majority of patients had combined NPGS with phaco-PCIOL (SCB group: 24/30 eyes; control group: 20/30 eyes); few patients had phacotrabeculectomy, NPGS, and trabeculectomy alone (Table 1). 

In the control group, no one received SCB as part of their postoperative management. Eight eyes from the control group did not undergo any intervention during their postoperative period. The rest of the control group (22/30 eyes) underwent one or a combination of the following treatments: 5-FU injection (12/30 eyes), suture lysis (9/30 eyes), goniopuncture (11/30 eyes), needling (2/30 eyes), and subconjunctival injection of triamcinolone acedonide (1/30 eyes). Patients from the SCB group received similar pattern of postoperative management, but more patients underwent needling compared to those of the control group: 5-FU injection (10/30 eyes), suture lysis (7/30 eyes), goniopuncture (8/30 eyes), needling (5/30 eyes), and subconjunctival injection of triamacinolone (2/30 eyes) and betamethasone (1/30 eyes). Sixteen eyes of the SCB group received only bevacizumab as their postoperative management with no other intervention required. Compared to the control groups, the SCB group had fewer patients who required different types of interventions: 14/30 eyes in the SCB group versus 22/30 eyes in the control group (Table 2).

IOP was reduced from 21.9 (±9.8) mmHg preoperatively to 11.9 (±4.7) mmHg on the last follow-up visit in the control group. In the SCB group, the average IOP was reduced from 19.6 (±8.9) mmHg preoperatively to 14.0 (±4.7) mmHg on the last visit. The number of IOP-lowering medications was reduced from 3.1 (±1.0) to 0.4 (±0.8) in the control group and from 3.0 (±1.1) to 0.7 (±1.1) in the SCB group. The reductions in number of medications and IOP were statistically significant in both groups (*P* < 0.001). The mean final IOP was slightly higher in the SCB group (14.0 ± 4.7 mmHg) compared to that of the control group (11.9 ± 4.7 mmHg); however the difference was not statistically significant (*P* = 0.36). In fact, there were no statistical differences in final IOP, the number of medications or reductions in IOP between the SCB and control groups (Tables 3 and 4). 

The complications observed in the control group included one case of blebitis requiring topical antibiotic treatment, two cases of iris incarceration, and one case of bleb leak requiring medical treatment. In the SCB group, three patients developed branch retinal vein occlusion (BRVO) during their follow-up periods, two patients had choroidal detachments, and one patient had a bleb leak that required surgical repair. The three cases of BRVO were all patients with POAG. Two of the three patients were not known for any systemic risk factors such as hypertension, diabetes, or atherosclerosis, whereas the third patient with BRVO had had central retinal vein occlusion in the past (Table 5).

## 4. Discussion

Studies have shown that anti-VEGF agents could modulate wound healing through antifibroblast effect in addition to its antiangiogenic role [11]. VEGF receptors have been found on Tenon fibroblasts [12]. In vitro cell cultures have also demonstrated that the application of bevacizumab inhibited Tenon fibroblasts proliferation, reduced collagen deposition, and induced apoptosis of fibroblasts [6, 13]. In vivo animal studies in rabbits undergoing trabeculectomy have demonstrated the efficacy of bevacizumab in prolonging bleb survival compared to 5-FU injection [14]. One study has also shown that the bleb survival rate was the highest in the rabbits receiving a combination of subconjunctival 5-FU and bevacizumab injections (100% survival rate) compared to the rabbits receiving only one of the two injections (5-FU: 25% survival rate or bevacizumab: 50% survival rate), suggesting that a combination of antimetabolite and anti-VEGF might be superior to the use of either agent alone [15]. Such synergetic effect of antimetabolite and anti-VEGF has been reported in clinical trials involving patients treated for colorectal cancer where delayed wound healing was observed in the group treated with systemic bevacizumab and 5-FU compared to the group treated with 5-FU-based chemotherapy alone [16]. Clinical studies on the combined use of anti-VEGF and antifibrotic agents are limited to the use of ranibizumab. In a prospective study, Kahook compared the effect of combined intravitreal injection of ranibizumab and topical MMC to MMC alone in patients undergoing trabeculectomy. His study found that patients receiving a combination of MMC and anti-VEGF demonstrated more diffuse bleb, but both groups had the same final IOP [17]. Our comparative series attempted to illustrate the combined use of SCB in addition to other postoperative interventions including the use of antimetabolites such as MMC and 5-FU. We also did not find significant advantage in terms of the final IOP value or the reduction in medications. Unfortunately, the difference in bleb morphology between the SCB and control groups could not be compared due to lack of documentation; whether the addition of bevacizumab would influence the bleb morphology could not be determined in this series. 

**Figure 1:**
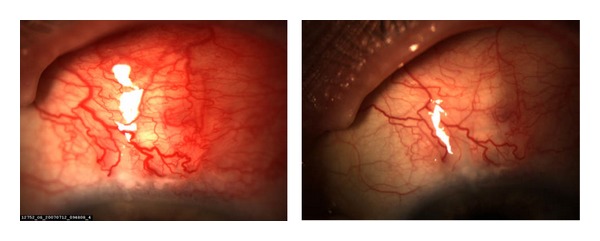
Reduction in the number of vessels and the vessel diameter after a single subconjunctival injection of bevacizumab.

The optimal timing and delivery method of bevacizumab in GFS are yet to be determined. Pharmacokinetics studies in rabbits have shown that both subconjunctival and intravitreal administrations of bevacizumab reached adequate intraocular concentration, whereas the topical route did not [18]. Topical use of anti-VEGF is, however, sufficient in regressing corneal neovascularization. It is debatable if the topical administration of anti-VEGF will be sufficient in glaucoma surgery as the target place is the subconjunctival space. Topical administration has the advantage of less systemic absorption but requires frequent dosing, whereas a single intravitreal or subconjunctival injection may be sufficient as the duration of bevacizumab can be detected at least up to two weeks in aqueous humour [18]. The duration of injection will cover the peak inflammatory and healing phase that occurs for about one week after filtering surgeries [11]. In the prospective studies on anti-VEGF use in GFS, subconjunctival injection is the most commonly used route except in one study that used intravitreal injection [8–10, 17]. Most of studies also used a single injection intraoperatively or postoperatively. Only the study by Sengupta and associates used three subconjunctival injections [9]. There is no study comparing a single use of bevacizumab to repeated injections. In our study, SCB was given postoperatively when the bleb appeared to be hypervascular. This approach is reasonable as newly formed vessels depend on VEGF to survive, and the inhibition of VEGF will result in regression of neovascularization [19]. Clinically, decreased bleb vascularity has been observed after one single injection of bevacizumab into the bleb in some case reports as well as in our series of patients (Figure 1). In our opinion, intravitreal or subconjunctival injection may not differ much in the management of GFS, as both methods achieve adequate aqueous concentration and have similar pharmacokinetics. Intravitreal injection has the additional ocular risks of inflammation, retinal detachment, and endophthalmitis. One ongoing study is investigating the use of topical ranibizumab four times a day for one month in postoperative period for trabeculectomy; its results may illustrate if topical treatment will be also an option in the management of glaucoma surgery [20].

We observed three cases of branch retinal vein occlusion (BRVO) in the group of 28 patients receiving bevacizumab. This high-incidence BRVO is probably coincidental since the onset of BRVO was seven to eight weeks after the injection of bevacizumab. Other series on glaucoma filtering surgery did not report any vascular complication related to the use of anti-VEGF agents [8, 9, 17]. However, BRVO is a serious ocular complication, and we should still be cautious about the possible vascular side effects of anti-VEGF agents. Systemic use of bevacizumab is associated with various vascular events such as hypertension, hemorrhage, and thromboembolism [21]. Regarding the ocular use of bevacizumab, rare events such as retinal ischemia, central retinal artery occlusion, subretinal hemorrhages, and systemic complications such as elevated blood pressure, stroke, and myocardial infarctions have also been reported in surveys and retrospective studies [22, 23]. Pooled results from clinical trials have suggested a higher incidence of stroke in patients receiving a higher dose of ranibizumab (0.5 mg) compared to that in those receiving a lower dose (0.3 mg) [24]. Some studies have also shown that OAG patients had elevated VEGF levels in their aqueous humour and plasma compared to normal controls [25, 26]. One of VEGF functions includes vasodilation by inducing the synthesis of nitric oxide. The presence of VEGF is likely important for normal function and repair of vascular endothelium, and the blockage of VEGF may lead to endothelial dysfunction [19, 25]. Although there is no definitive evidence suggesting that glaucoma patients are prone to side effect of anti-VEGF agents, as their use may be more frequent in glaucoma patients, the safety profile of anti-VEGF agents should be followed.

In conclusion, our comparative series did not found that subconjunctival injection of bevacizumab had additional benefit in term of IOP reduction. The bleb morphology could not be assessed in this series due to the retrospective nature of the study and lack of bleb description in every patient. Whether bevacizumab will influence the bleb morphology and whether this influence will be clinically relevant need to be addressed by prospective studies with longer follow-up time. In our opinion, subconjunctival and intravitreal injections of anti-VEGF would have the same efficacy as the pharmacokinetics of either delivery route will cover the inflammatory phase after filtering surgeries [18]. Of significance, there were three cases of BRVO observed in the bevacizumab group. Clinicians should monitor potential side effects of anti-VEGF agents as their safety profile in glaucoma patients may not be the same as that in AMD patients.

## Figures and Tables

**Table 1:** Characteristics of the subconjunctival bevacizumab (SCB) and control groups.

	SCB group	Control group	P value
Age (years)	71.5 ± 9.2	73.9 ± 7.4	0.21
Gender			
Male	12	13	0.78
Female	16	15	
Ethnicity			
Caucasian	26 (92.8%)	27 (96.4%)	
African decent	1 (3.6%)	1 (3.6%)	—
Asian	1 (3.6%)	0	
Total patients	28	28	
Eye			
Right	19	17	0.60
Left	11	13	
Total eyes	30	30	
Diagnosis			
Open-angle glaucoma	26 (86.7%)	25 (83.3%)	
Ocular hypertension	2 (6.7%)	2 (6.7%)	0.90
Others (pigmentary, pseudoexfoliation)	2 (6.7%)	3 (10%)	
Total eyes	30	30	
Surgery types			
Phaco-NPGS	20 (66.7%)	24 (80%)	
Phacotrabeculectomy	6 (20%)	3 (10%)	—
NPGS	3 (10%)	2 (6.7%)	
Trabeculectomy	1 (3.3%)	1 (3.3%)	
Total eyes	30	30	
Preoperative IOP (mmHg)	19.6 ± 8.9	21.9 ± 9.8	0.51
Preoperative number of medications	3.0 ± 1.1	3.1 ± 1.0	0.48
Mean follow-up time (months)	16.9 ± 8.2	19.6 ± 11.5	0.16

**Table 2:** Different postoperative interventions in the subconjunctival bevacizumab (SCB) and control groups.

	SCB group *N* = 30	Control group *N* = 30
No postoperative intervention	0	8

SCB as the only postoperative intervention	16	0
Subconjunctival 5-FU injection	10	12
Needling (± 5-FU)	5	2
Suture lysis	7	10
Goniopuncture	8	11
Subconjunctival injection of triamcinolone acetonide	2	1
Subconjunctival injection of betamethasone	1	0

**Table 3:** Mean IOP in the subconjunctival bevacizumab (SCB) and control groups during the follow-up period.

	SCB group *N* = 30	Control group *N* = 30	*P* value
IOP 1 month (mmHg)	14.4 ± 4.7	12.8 ± 4.8	0.83
IOP 3 months (mmHg)	11.8 ± 4.6	12.8 ± 5.0	0.44
IOP 6 months (mmHg)	11.5 ± 3.7	12.5 ± 4.3	0.42
IOP 1 year (mmHg)	13.8 ± 3.6	13.2 ± 4.9	0.30
IOP final visit (mmHg)	14.0 ± 4.7	11.9 ± 4.7	0.36

**Table 4:** Comparison in the reduction of IOP and medications in both subconjunctival bevacizumab (SCB) and control groups.

	SCB group *N* = 30	Control group N = 30	P value
Final IOP (mmHg)	14.0 ± 4.7	11.9 ± 4.7	0.36
Percentage of IOP reduction	20%	36%	0.73
Final number of medications	0.7 ± 1.1	0.4 ± 0.8	0.11
Percentage of the reduction of medication use	32%	27%	0.15

**Table 5:** Characteristics of patients with branch vein occlusion (BRVO) in the subconjunctival bevacizumab (SCB) group.

Patient	Age (years)	Sex	Medical history	Eye	Diagnosis	Preoperative VA	Type of surgery	Postoperative interventions	Onset of BRVO	Treatment	Final VA	Final IOP (mm Hg)
1	73	F	None	OS	POAG	20/40	Phacotrab.	Needling goniopuncture suture lysis SCB	14.8 weeks postsurgery 8.8 weeks after SCB	Focal laser	20/30	8

2	81	M	HTN BRVO OD	OD	POAG	20/50	Phaco-NPGS	SCB goniopuncture suture lysis	7.8 weeks postsurgery 6.8 weeks after SCB	IVTA	20/150	10

3	80	M	Hyperchol.	OS	POAG	20/40	Phacotrab.	SCB	9.7 weeks postsurgery 8.3 weeks after SCB	Focal laser	20/40	14

HTN: hypertension; hyperchol.: hypercholesterolemia; POAG: primary open-angle glaucoma; phacotrab.: phacotrabeculectomy; NPGS: nonpenetrating glaucoma surgery; SCB: subconjunctival injection of bevacizumab; IVTA: intravitreal injection of triamcinolone acetonide; VA: visual acuity.
